# Hypophosphites in the Toothache of Pregnancy

**Published:** 1872-06

**Authors:** 


### Hypophosphites in the Toothache of Pregnancy.

Dr. Sterling believes that the toothache so common in pregnancy, results from the abstraction from the blood of the salts requisite for the construction of the bones of the fœtus; and accordingly recommends 1½ grains of hypophosphite of lime, soda, potash, and manganese, daily.—*American Journal*, New Series, cxiii.—*London Practitioner* (Baltimore Reprint.)

